# The ten-year risk of developing cardiovascular disease among public health workers in North-Central Nigeria using Framingham and atherogenic index of plasma risk scores

**DOI:** 10.1186/s12889-022-13044-9

**Published:** 2022-04-27

**Authors:** Olubunmi Abiola Olubiyi, Bosede Folashade Rotimi, Munirat Ayoola Afolayan, Bilqis Wuraola Alatishe-Muhammad, Olufemi Mubo Olubiyi, Ahmed Dahiru Balami

**Affiliations:** 1 Department of Disease Control and Elimination, Medical Research Council Unit The Gambia at the London, School of Hygiene and Tropical Medicine, Atlantic Boulevard, Fajara. P.O Box 273, Banjul, The Gambia; 2 Department of Community Health, Federal Medical Centre, Bida, P.M.B 14 Niger State Nigeria; 3 Department of Community Health, Nigerian Navy Reference Hospital, Ojo, Lagos State Nigeria; 4 Department of Planning and Statistics, Kwara State Ministry of Health, Ilorin, Kwara State Nigeria; 5 Department of Family Medicine, Bafrow Medical Centre, 156 Mosque Rd, Serrekunda, Banjul, The Gambia; 6 Department of Clinical Research, London School of Hygiene and Tropical Medicine, Keppel Street, London, WC1E 7HT UK

**Keywords:** Cardiovascular disease, Framingham risk, Atherogenic index, Health workers, Symptoms, Risk factors

## Abstract

**Background:**

Estimation of total cardiovascular disease (CVD) risk with the use of risk prediction charts such as the Framingham risk score and Atherogenic index of plasma score is a huge improvement on the practice of identifying and treating each of the risk factors such as high blood pressure and elevated blood cholesterol. The estimation of the total risk highlights that CVD risk factors occur together and thereby predicts who should be treated. There is scarcity of data on the risk scoring of adults in Nigeria including health workers. Therefore, this study was done to estimate the cardiovascular risks of health workers in public health services in north-central Nigeria.

**Methods:**

A cross-sectional survey was performed using validated Framingham risk score calculator and calculation of risk based on the lipid profile of 301 randomly selected health workers in North-central Nigeria. Descriptive analysis was done using frequency counts and percentages while inferential statistics were done using chi square and correlation analyses using statistical Package for Social Sciences (SPSS) version 21.0. The confidence level was 95% and the level of significance was set at 0.05.

**Results:**

The 10-year risk of developing CVD was generally low in the health workers. Using Framingham risk score, 98.3% of health workers have low risk, 1.0% have moderate risk and 0.7% have high risk. Among the cadres of health workers, 1.5% of the nurses have moderate risk while 2.5% of the doctors and 3.3% of the CHEWs have high risk of developing CVD in 10 years. Using Atherogenic index of plasma scoring, only 2% of the health workers have high risk, 4.7% have intermediate risk while 93.4% have low risk. Across the cadres, 6.3% of the nurses and 3.3% of the CHEWs have intermediate risk while 2.4% of the nurses and 3.3% of the CHEWs have high risk. These findings were however not statistically significant.

**Conclusions:**

The 10-year risk of developing cardiovascular disease was low in the health workers in this study using both Framingham’s risk score and atherogenic index of plasma scores.

**Supplementary Information:**

The online version contains supplementary material available at 10.1186/s12889-022-13044-9.

## Background

Cardiovascular disease (CVD) has become very common all over the world in both developing and developed nations, especially among adults [1]. In Sub-Saharan Africa, the incidence has been rising steadily for many years [2]. About a century ago, less than 10% of all-cause mortality were attributable to CVDs [3]. but currently, CVDs are responsible for about 30% of deaths worldwide [2, 3]. In 2012, about 17.5 million CVD deaths were recorded leading to about 46.2% of global NCD deaths [4]. About 80% of this mortality occurred in LMICs [2]. Statistics from the United States show that nearly 2,200 Americans die of CVDs daily, resulting in about 801,000 deaths per year [5], at an average of 1 death per 40 seconds [5]. In Nigeria, paucity of data has made it impossible to have baseline statistics on CVD mortality [6] but there is evidence of increasing rates of morbidity and mortality from risk factors of CVD [4]. Cardiovascular diseases include stroke, coronary heart disease, aortic aneurysms and dissection, deep vein thrombosis, pulmonary embolism, among others [6, 7].

Cardiovascular disease is not cause specific; it has both modifiable and non-modifiable risk factors. The morbidity and mortality from CVDs to a large extent is attributable to modifiable risk factors which were initially prevalent in the developed countries [1, 2]. The modifiable risk factors include but not limited to: physical inactivity, increased body mass index (BMI), high blood pressure, diabetes, high cholesterol level, tobacco use, and unhealthy diet including high salt intake [6, 8–10].

To assess the prevalence of cardiovascular risk, there are certain tests and behavioural factors to be considered. These also predict the likelihood of having CVD and determine whether the degree of risk is mild, moderate or severe [1, 11–13]. The assessment of CVD risk factors is done by taking history about behaviours and taking physical and biochemical measurements which are as a result of the individual’s behaviours.

In developed countries, the risk assessment methods used are effective but costly [13]. However, these methods may not be possible in low income countries [13]. Currently used in developing countries are CVD risk management tools developed by the World Health Organization (WHO). Many studies done in Nigeria usually focus only on anthropometric and biological estimation of risks [1, 12, 14, 15]. Estimation of total CVD risk with the use of risk prediction charts is a huge improvement on the practice of identifying and treating each of the risk factors such as high blood pressure and elevated blood cholesterol. The estimation of the total risk highlights that CVD risk factors occur together and thereby predicts who should be treated. An example of the risk score calculator is that used in the Framingham Heart Study [16].

One of the levels of prevention involves early diagnosis and prompt treatment of risk factors of CVD and this is done in people with high risk [17]. Screening methods used include physical measures such as weight and height check to determine the body mass index, fasting blood glucose for diabetes, fasting lipid profile for dyslipidaemia and blood pressure measurement for hypertension. Those with confirmed risks are then treated promptly and effectively [17]. Drugs have shown to be very effective in the management of CVD and its risk factors [17]. Early diagnosis and prompt treatment of cases has been shown to reduce mortality from stroke by 45% [17].

Estimation of risk of developing CVD can also be by the Framingham risk score chart and atherogenic index of plasma score. The Framingham risk score chart which estimates the risk of developing CVD [18, 19] consists of seven variables [20]. The variables are age, gender, total cholesterol, high density lipoproteins (HDL) cholesterol, smoking history, systolic blood pressure, diabetes mellitus as well as the current use of medication for the treatment of high blood pressure [20, 21]. The variables after computation into an application grades the risks as follows: low risk (Risk < 10%), moderate risk (Risk 10% to < 20%), and high risk (Risk ≥ 20%) [19].

Similarly, the atherogenic index of plasma (AIP) can also be used as an index for estimation CVD risk [22]. The logarithmic calculation of the ratio of serum level of triglycerides to high density lipoproteins (HDL-C) is used to determine AIP and it is a better diagnostic tool than ordinary lipid profile results [22]. When individuals have deranged lipid profiles, they become prone to atherosclerosis and its complications.

Health workers are a major group of professionals in the class of essential services all over the world [23]. Their work determines the health of the society at large, therefore, they are critical to the maintenance of a healthy society. They work in both public and private health services and offer services in primary, secondary and tertiary health care facilities and research institutes. Health workers comprise of doctors, nurses, laboratory scientists and technicians, pharmacists and pharmacy technicians, community health extension workers and community health officers, radiographers, audiographers, nutritionists and other allied health workers.

The aim of the study was to describe and predicts the ten-year estimation of developing cardiovascular disease among health workers in public health services in north-central Nigeria using validated Framingham and atherogenic index of plasma scores. Due to poor data on risk estimation in Nigeria using Framingham and atherogenic index of plasma scores, this study will provide baseline data for which further studies will be done.

Also, very few studies have been done among health workers in Nigeria. It is generally assumed that health workers have optimum health and thereby are not studied. Unfortunately, there have been reports of sudden death in this population in recent times. Therefore, estimation of cardiovascular disease risk in this population will define the strategies for control in them.

## Methods

### Study design and population

The study was a cross-sectional study conducted in 2019 with data collected over a period of one month. A total of 301 health workers were randomly selected using multi-stage sampling technique. The inclusion criteria for the study were health workers who were trained in accredited institutions, working in public health facilities and who have spent a minimum of one year in service while the exclusion criteria were health workers with history of cardiovascular disease.

#### Data collection process and instruments

The study instruments used included: semi-structured self-administered questionnaire adapted from the WHO STEP-wise approach to surveillance (STEPS), stadiometer, sphygmomanometer and laboratory investigations for fasting lipid profile and fasting blood glucose, and Framingham risk score chart. The questionnaire includes sections on socio-demography, knowledge of CVD risks, CVD risk prevention practices. Validation of the questionnaire was done using face validity and content validity [24]. The anthropometric and blood pressure measurements as well as laboratory investigations were done using WHO recommended standard operating procedures and equipments. Each respondent’s weight was measured with light clothes on and bare feet using calibrated and standardized OMRON BF 400 weighing scale to the nearest kilogram (0.1 kg). The height of the respondents was also measured using the Leicester Stadiometer while standing in an erect position with the back against the wall. The respondents were measured without shoes and head gear or cap to the nearest 0.01 m (m). The BMI was calculated by dividing the weight (kg) by the square of the height (m^2^) and categorized according to WHO classification [25].

Blood pressure measurements was done using calibrated and standardized OMRON M6 Comfort Automatic Sphygmomanometer and re-calibrated daily and after 10 measurements. The blood pressure readings measured in mmHg were classified based on the JNC VII guidelines [26]. The total cholesterol was analyzed by GPO-PAP methodology [27, 28]. The triglyceride and HDL cholesterol were determined using the colorimetric assay while the LDL cholesterol was determined using the Friedewald’s formula, LDL cholesterol (mmol/L) = total cholesterol-HDL cholesterol-triacylglycerol/5 [29]. The results of the serum cholesterol were categorized [30].

### Data analysis

Data was collected over one month using the self-administered semi-structured questionnaire. Anthropometric and blood pressure measurements as well as blood samples for lipid profile and blood glucose following a 12-h fast were collected using lithium heparin bottles by research assistants.

All measurements were done according to WHO standards. Following analysis of the samples, Atherogenic index of plasma (AIP) was determined by using logarithmic transformation of the ratio of triglyceride to high density lipoprotein, Log (Tg/HDL-C) [31]. The AIP scores < 0.11, 0.11–0.24, and ≥ 0.24 were graded as low risk, intermediate and high risk respectively [22]. Also, the Framingham risk score calculator was used to estimate each health worker’s risk of developing CVD [18, 19]. The calculator is an application on Google playstore. The calculator utilizes the input of eight variables to arrive at a score [20]. These variables which score and predict an individual’s 10 year risk of developing CVD are age, gender, total cholesterol, HDL cholesterol, smoking history, systolic blood pressure, diabetes mellitus as well as the current use of medication for the treatment of high blood pressure [20, 21]. After computation, the scores were categorized as follows: low risk (Risk < 10%), moderate risk (Risk 10% to < 20%), and high risk (Risk ≥ 20%) [19].

The data was then analyzed using Statistical Package for Social Sciences (IBM/SPSS) version 21. Categorical variables are summarized as frequencies and percentages.. Chi-square test of association (including Fisher’s exact test and Yates corrected Chi-square where appropriate) was used to test for association between clinical risk category and gender, cadre, knowledge and practice of the health workers and Spearman’s correlation coefficient was used to determine the correlation between AIP and CVD risk factors. A confidence interval of 95% was used in this study and a *p* value of < 0.05 was considered as significant.

## Results

The ages of the respondents ranged between 21–58 years with a mean age (± SD) of 39.3 (± 8.30) years. More than half, 160 (53.2%) of the respondents were females. About two-thirds of the participants, 205(68.1%) were nurses and 201 (66.8%) work at the tertiary institution. Majority of the participants have either diploma or bachelors’ degree (42.9% respectively). The median income and interquartile range (IQR) in Naira per month was **₦**152,000 (**₦**100, 000–250,000). ( Table 1).

**Table 1:** Socioeconomic characteristics of the health workers

Socioeconomic characteristics	Frequency (*N* = 301)	%
**Age (years)**		
21 – 30	54	17.9
31 – 40	115	38.3
41 – 50	100	33.2
51 – 60	32	10.6
Mean (± SD)	39.30 (± 8.30)	
Range	22 – 58	
**Sex**		
Male	141	46.8
Female	160	53.2
**Cadre**		
Doctor	41	13.6
Nurse	205	68.1
Pharmacist	9	3.0
CHEW/CHO	30	10.0
Laboratory Scientist/tech	16	5.3
**Health Facility**		
PHC	27	9.0
Secondary	73	24.2
Tertiary	201	66.8
**Level of education**		
Diploma	129	42.9
Bachelors	129	42.9
Postgraduate	43	14.2
**Income (₦)**		
≤ 100,000	80	26.6
101,000—200,000	128	42.5
201,000—300,000	60	19.9
> 300,000	33	11.0
Median	152,000.00	
Interquartile range	100,000.00 – 250,000.00	

The age of the respondents ranged between 21–58 years with a mean age of 39.3 years while the modal age group was 31–40 years. More than half, 160 (53.2%) of the respondents were females

About two-thirds of the participants, 205(68.1%) were nurses and 201 (66.8%) work at the tertiary institution. Majority of the participants have either diploma or bachelors’ degree (42.9% respectively). The median income in Naira per month was **₦**152,000 with an interquartile range of **₦**100, 000–250,000

The 10-year risk of developing cardiovascular disease among the health workers using Framingham risk score shows that only 0.7% of them have high risk, 1.0% have moderate risk, while 98.3% have low risk. Therefore, majority of the health workers have a low 10-year risk of developing cardiovascular disease. Likewise, using Atherogenic Index of Plasma scoring, 2% have high risk, 4.7% have intermediate risk, while 93.4% have low risk. (See Table 2). This also means that majority of the health workers have mild risk of developing CVD from dyslipidaemia.

**Table 2:** Framingham and Atherogenic Index of Plasma Risk score grading of the health workers

Risk scoring	Frequency (*N* = 301)	%
**Framingham risk score**		
Low risk	296	98.3
Moderate risk	3	1.0
High risk	2	0.7
**Atherogenic Index of Plasma**		
Mild risk	281	93.4
Intermediate	14	4.7
High risk	6	2.0

Following the grading of the Framingham risk scores, majority of the health workers, 296 (98.3%) have low 10-year risk of developing cardiovascular disease. Likewise, after grading the Atherogenic Index of Plasma scores, majority of the health workers, 281 (93.4%) have low risk of developing CVD from dyslipidaemia

**Fig. 1:**
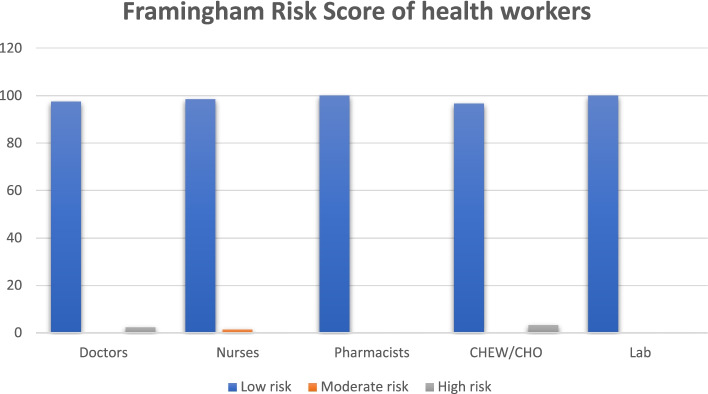
Framingham risk score of the health workers

Among the different cadres of health workers, 97.5% of the doctors, 98.5% of the nurses, 100% of the pharmacists, 96.7% of the CHEWs and 100% of the laboratory scientists/technicians had low 10-year risk of developing CVD using Framingham risk score. However, 1.5% of the nurses had moderate risk while 2.5% of the doctors and 3.3% of the CHEWs had high risk of developing CVD in 10 years. (See Fig. 1). Using AIP scores, 100% of the doctors, 91.3% of the nurses, 100% of the pharmacists, 93.4% of the CHEWs and 100% of the laboratory scientists/technicians had low risk of AIP dyslipidaemia. However, 6.3% of the nurses and 3.3% of the CHEWs had intermediate risk while 2.4% of the nurses and 3.3% of the CHEWs had high risk. These findings were however not statistically significant. (See Table 3).

**Table 3:** Relationship between the lipid profile and Atherogenic index of plasmascores of the health workers and job cadre

	**Job cadre**							
	**Doctor**	**Nurse**	**Pharm**	**CHEW**	**Lab**	**Total**	**χ**^**2**^	***p* value**
**Variable**	**n (%)**	**n (%)**	**n (%)**	**n (%)**	**n (%)**	**N**		
**T.C**								
Optimal	15(36.6)	69(33.7)	2(22.3)	16(53.3)	10(62.5)	112(37.2)	11.235^Y^	0.188
Borderline	15(36.6)	80(39.0)	3(33.3)	4(13.4)	2(12.5)	104(34.6)		
High risk	11(26.8)	56(27.3)	4(44.4)	10(33.3)	4(25.0)	85(28.2)		
**HDL**								
High risk	1(2.4)	16(7.8)	0(0.0)	2(6.7)	3(18.8)	22(7.3)	4.128^Y^	0.845
Beneficial	2(4.9)	21(10.2)	1(11.1)	2(6.7)	0(0.0)	26(8.6)		
**LDL**								
Optimal	30(73.2)	150(73.2)	8(88.8)	21(70.0)	12(75.0)	221(73.4)	3.199^Y^	0.999
Borderline	6(14.6)	26(12.7)	1(11.1)	6(20.0)	2(12.5)	41(13.6)		
High risk	5(12.2)	29(14.2)	0(0.0)	3(10.0)	2(12.5)	40(13.0)		
**Triglyceride**								
Optimal	38(92.7)	181(88.3)	9(100.0)	26(86.7)	16(100.0)	270(89.7)	1.458^Y^	0.993
Borderline	1(2.4)	15(7.3)	0(0.0)	3(10.0)	0(0.0)	19(6.3)		
High risk	2(4.9)	9(4.4)	0(0.0)	1(3.3)	0(0.0)	12(4.0)		
**AIP**								
Mild risk	41(100.0)	187(91.3)	91(100.0)	28(93.4)	16(100.0)	281(93.4)	3.160Y	0.923
Intermediate	0(0.0)	13(6.3)	0(0.0)	1(3.3)	0(0.0)	14(4.7)		
High risk	0(0.0)	5(2.4)	0(0.0)	1(3.3)	0(0.0)	6(2.0)		

*χ*^*2*^Chi square test, *Y* Yates corrected Chi square

^*^p value < 0.05, *Pharm* Pharmacists, *Lab* Laboratory scientist/technician

There was no statistically significant association between the fasting lipid profile as well as the atherogenic index of plasma of the health workers and their job cadre

Nearly all those with low risk (97%) had good knowledge of CVD risk factors using Framingham’s risk score grade. Also, majority (96.8%) of those with mild AIP dyslipidaemia risk had good knowledge. (See Table 4). Only 57 (19.3%) health workers with low Framingham 10-year risk of developing CVD had good practice. Also, 56 (19.9%) of those with mild AIP dyslipidaemia risk had good practice. However, these were not statistically significant. (See Table 5. There was no gender disparity in the risk estimation of the health workers as there was no statistically significant association between sex, Framingham risk score and atherogenic index of plasma (AIP) score. (See Table 6).

**Table 4:** Relationship between knowledge of cardiovascular disease risk and clinical risk scoring

	**Knowledge**				
**Clinical risk scoring**	**Good**	**Poor**	**Total**	**χ**^**2**^	***p* value**
	**n (%)**	**n (%)**	**N**		
**Framingham risk score grade**					
Low risk	287 (97.0)	9 (3.0)	296	5.289^Y^	0.071
Moderate risk	3 (100.0)	0 (0.0)	3		
High risk	2 (100.0)	0 (0.0)	2		
**Atherogenic Index of Plasma**					
Mild risk	272 (96.8)	9 (3.2)	281	0.608^Y^	0.738
Intermediate	14 (100.0)	0 (0.0)	14		
High risk	6 (100.0)	0 (0.0)	6		

*χ*^*2*^Chi square test, *Y* Yates corrected Chi square

There was no statistically significant association between good knowledge of cardiovascular disease and Framingham risk score and AIP dyslipidaemia risk score. (*p* > 0.05)

**Table 5:** Relationship between practice of cardiovascular disease prevention and clinical risk

	**Practice**					
**Clinical risk scores**	**Poor**	**Fair**	**Good**	**Total**	**χ**^**2**^	***p*-value**
	**n (%)**	**n (%)**	**n (%)**	**N (%)**		
**Framingham**						
Low risk	37 (12.5)	202 (68.2)	57 (19.3)	296 (98.3)	0.474^Y^	0.976
Moderate risk	0 (0.0)	2 (66.7)	1 (66.7)	3 (1.0)		
High risk	0 (0.0)	1 (50.0)	1 (50.0)	2 (0.7)		
**Atherogenic Index of Plasma**						
Mild risk	34 (12.1)	191 (68.0)	56 (19.9)	281 (93.4)	0.261^Y^	0.992
Intermediate	2 (14.3)	10 (71.4)	2 (14.3)	14 (4.7)		
High risk	1 (16.1)	4 (66.7)	1 (16.7)	6 (2.0)		

*χ*^*2*^Chi square test, *Y* Yates corrected Chi square

There was no significant relationship between good CVD prevention practices and clinical risk scoring. (*p* values > 0.05)

**Table 6:** Relationship between sex and clinical risk of the health workers

	**Sex**				
	**Male**	**Female**	**Total**	**χ**^**2**^	***p* value**
**Variable**	**n (%)**	**n (%)**	**N (%)**		
**Framingham risk score**					
Low risk	137 (97.2)	159 (99.4)	296 (98.3)	3.293^F^	0.176
Moderate risk	3 (2.1)	0 (0.0)	3 (1.0)		
High risk	1 (0.7)	1 (0.6)	2 (0.7)		
**AIP**					
Mild risk	130 (92.2)	151 (94.4)	281 (93.4)	3.171^F^	0.210
Intermediate risk	6 (4.3)	8 (5.0)	14 (4.6)		
High risk	5 (3.5)	1 (0.6)	6 (2.0)		

*χ*^*2*^Chi square test, *F* Fisher’s exact test, *t* Independent Samples T test

There is no statistically significant association between sex Framingham risk score and atherogenic index of plasma (AIP) score

**Fig. 2:**
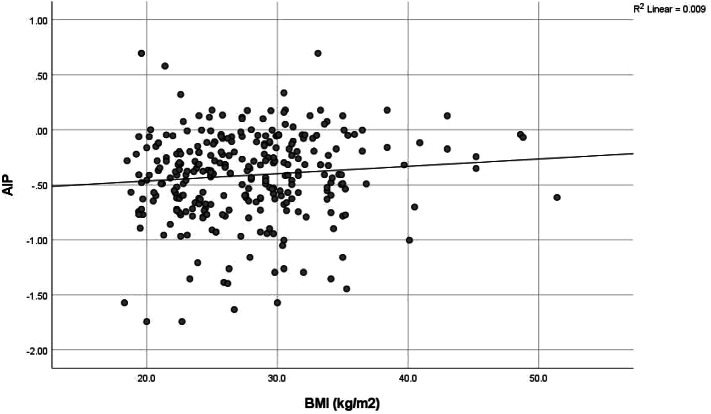
Correlation between AIP and BMI. There was a weak positive correlation between AIP and BMI though not strong (*r* = 0.118, *p* value 0.041). This was statistically significant

**Fig. 3:**
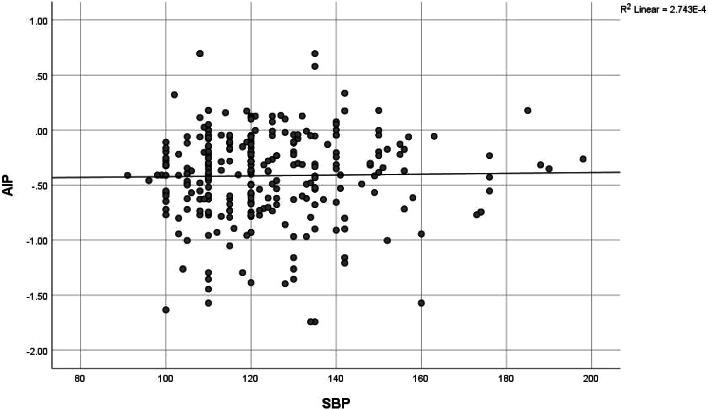
Correlation between AIP and systolic blood pressure. There was no correlation between AIP and systolic blood pressure (*r* = 0.043, *p* value 0.459)

**Fig. 4:**
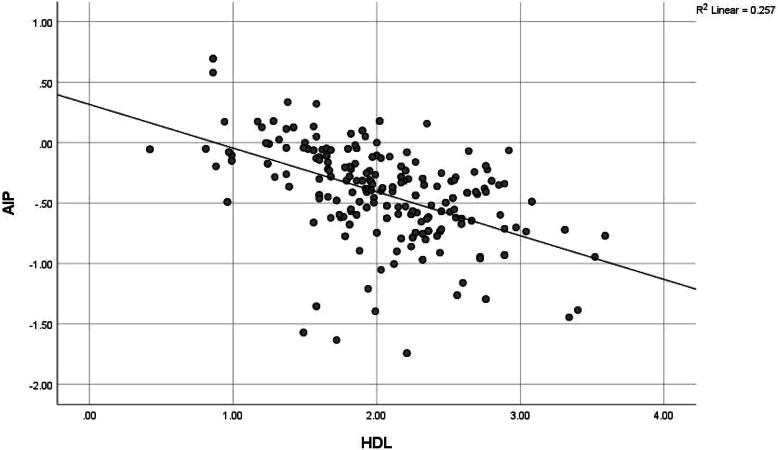
Correlation between AIP and HDL cholesterol. There was a strong negative correlation between AIP and HDL cholesterol (*r* = -0.558, *p* value < 0.001). The correlation was statistically significant

**Fig. 5:**
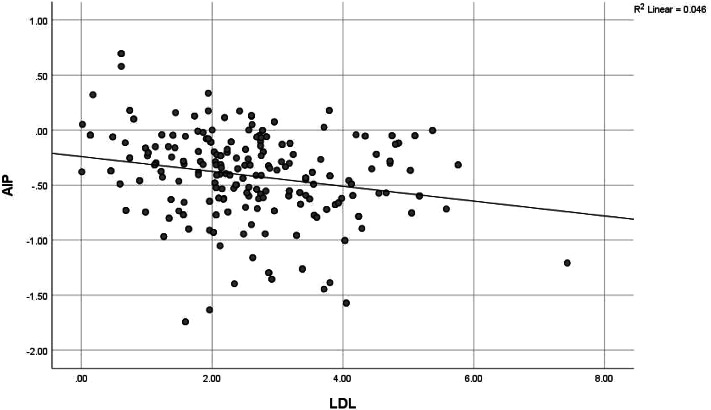
Correlation between AIP and LDL cholesterol. There was a weak negative correlation between AIP and LDL cholesterol (*r* = -0.215, *p* value < 0.001). The correlation was statistically significant

**Fig. 6:**
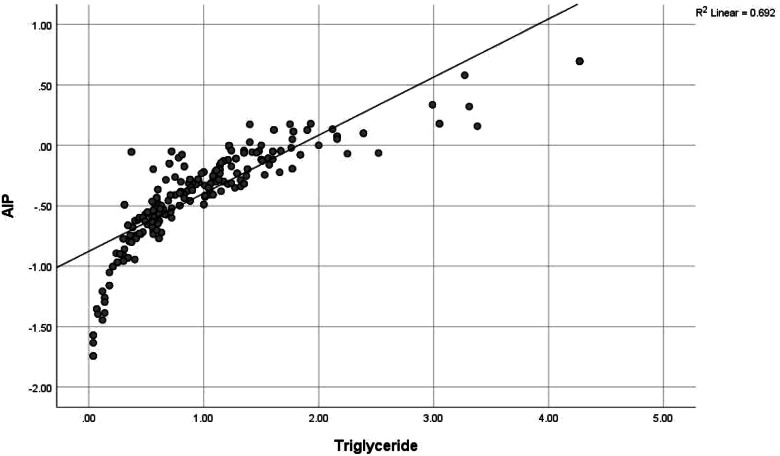
Correlation between AIP and triglyceride. There was a very strong positive correlation between AIP and triglyceride (*r* = 0.912). The correlation was statistically significant at *p* value < 0.001

**Fig. 7:**
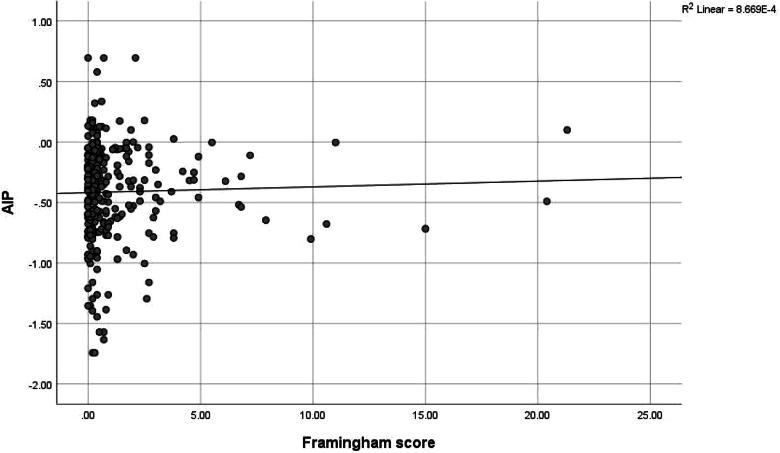
Correlation between AIP and Framingham risk score. There was no correlation between AIP and Framingham risk score (*r* = 0.011, *p* value 0.851)

Although only 20 (6.7%) of the health workers had intermediate-high risk AIP dyslipidaemia, there was a positively higher correlation between AIP score and triglyceride (0.912) and this was significant at *p* value < 0.001, while there was a negatively high correlation between AIP score and HDL cholesterol (-0.558) at *p* value of < 0.001. AIP risk was also significantly positively correlated to BMI (0.118, *p* value 0.041), waist circumference (0.174, *p* value 0.002) and fasting blood glucose (0.182, *p* value 0.002); and negatively correlated to LDL cholesterol (-0.215, *p* value < 0.001). (See Table 7 and Figs. 2,3, 4, 5, 6, 7).

**Table 7:** Correlation between Atherogenic Index of Plasma scores and CVD risk factors of respondents

	**AIP**	
**Risk factors**	**r**	***p* value**
BMI	0.118	0.041*
Blood pressure	-0.001	0.991
SBP	0.043	0.459
DBP	-0.014	0.815
Waist circumference	0.174	0.002*
Total cholesterol	-0.028	0.627
HDL	-0.558	< 0.001*
LDL	-0.215	< 0.001*
Triglyceride	0.912	< 0.001*
Fasting blood glucose	0.182	0.002*
Framingham score	0.011	0.851

*r* Spearman’s correlation coefficient rho

^*^p value < 0.05

Although only 20 (6.7%) of the health workers had intermediate-high risk AIP dyslipidaemia, there was a positively higher correlation between AIP score and triglyceride (0.912) and this was significant at *P* value < 0.001, while there was a negatively high correlation between AIP score and HDL cholesterol (-0.558) at *p* value of < 0.001. AIP risk was also significantly positively correlated to BMI (0.118, *p* value 0.041), waist circumference (0.174, *p* value 0.002) and fasting blood glucose (0.182, *p* value 0.002); and negatively correlated to LDL cholesterol (-0.215, *p* value < 0.001)

## Discussion

The study included respondents from a young population with mean age and standard deviation of 39.30 (± 8.30) years. This is similar to the study among health workers in Ghana (, mean age:32.1 ± 8.9 years) [23]. About 56.1% of them were young, between age 21–40 years. About 56.1% of participants were young, between 21–40 years possibly a reflection of the working population.This was lower than that reported in Ghana with the young population being 86.61% [23]. More than half (53.2%) of the health workers were females, a reflection of high nurses’ population in the study. This is consistent with other studies citing females being the dominant gender among nurses [32, 33]. This may also be due to the caring nature of women generally.

Two thirds of the participants, work in tertiary facility. This was probably because the tertiary institution had the highest population of health workers in the study area. The median monthly income was ₦152,000 (US$389 0.30). The interquartile range of monthly income was ₦100,000–250,000 (US$256–640). This is consistent with the finding from a survey of the Nigerian middle class with earning between US$480–645 [34]. This indicates that than an average Nigerian health worker should be able to afford basic amenities such as food and shelter [34].

The 10-year risk of developing cardiovascular disease was low among the health workers. Majority (98.3%) of the respondents had low risk while only 0.7% had high risk using the Framingham risk score. This is similar to the findings from the study among office workers in Iran in which 90.5% of the participants had low risk [35]. There was also no gender disparity in the Framingham risk estimation of the study participants as 99.4% of females and 97.2% of males had low risk. This is probably due to the population studied being young and knowledgeable in CVD risk prevention. This is a contrast to the study in Iran in which there was a significant higher risk in males than females [35]. Across the cadres of health workers, 97.5% of the doctors, 98.5% of the nurses, 100% of the pharmacists, 96.7% of the CHEWs and 100% of the laboratory scientists had low risk while only 1.5% of the nurses had moderate risk and 2.5% of the doctors and 3.3% of the CHEWs had high risk.

Atherogenic index of plasma (AIP) is an important marker for plasma atherogenicity which is used to predict CVD risk [31]. In this study, 93.4% have mild risk, 4.7% have intermediate risk while 6% have high risk. Females have higher AIP scores than males which means that females have higher risk of CVD dyslipidaemia risk factors than males. This may be due to the sedentary nature of many women. This is in contrast to studies which reports that premenopausal females are protected and have lower risk of CVD due to oestrogen [31, 36]. Furthermore, this study revealed that there was a statistically significant positive correlation between AIP and BMI (*r* = 0.118, *p* value 0.041), waist circumference (*r* = 0.174, *p* value 0.002), triglyceride (*r* = 0.912, *p* value < 0.001) and fasting blood glucose (*r* = 0.182, *p* value 0.002). This means that health workers with generalized obesity, visceral obesity, triglyceride dyslipidaemia and diabetes had high risk of AIP dyslipidaemia. There was also a statistically significant negative correlation between AIP and HDL (*r* = -0.558, *p* value < 0.001) and low density lipoproteins (LDL) cholesterol (*r* = -0.215, *p* value < 0.001). Therefore, health workers with high HDL and LDL cholesterol had low risk of AIP dyslipidaemia. This is corroborated by the findings in a study done among staff of a University in Malaysia which reported significant positive correlation between AIP and triglyceride (0.84, *p* < 0.05); and negative correlation between AIP and HDL cholesterol (-0.72, *p*< 0.05) with higher risks in females than males [22].

On the contrary, in an adult population in Iran, AIP risks were higher in males than females (*r* = -0.18, *p*< 0.001) [31]. It also reported statistically significant positive correlation reported between AIP and triglyceride (*r* = 0.77, *p* < 0.001), LDL cholesterol (*r* = 0.29, *p* < 0.001), total cholesterol (*r* = 0.2, *p* < 0.001), fasting blood glucose (*r* = 0.14, *p* < 0.001) and both systolic (*r* = 0.13, *p* < 0.001) and diastolic blood pressures (*r* = 0.16, *p* < 0.001) with a negative correlation to HDL cholesterol (*r* = -0.72, *p*< 0.001) [31]. The study also reported majority of the population to have high AIP risk [31]. Although this study reports only 6% high risk of AIP dyslipidaemia, there is a need for this group of people to continually test for dyslipidaemia especially with the high prevalence of overweight and obesity.

### Strength

The use of a semi-structured questionnaire is a strength as healthcare workers understood the terms which made correct interpretation of the questions easy.

### Limitation

The limitation with the study was the design (cross-sectional study) which made it impossible to determine the temporal relationship between the study variables. The use of semi-structured questionnaire was also a limitation as the healthcare workers could over report because of their knowledge of CVD risk factors.

## Conclusions

The 10-year risk of developing cardiovascular disease among health workers using Framingham and atherogenic risk scores was low in majority of the respondents probably because of their access to information regarding cardiovascular health. This study is offering a baseline data on the estimation of cardiovascular risk among health workers in North-central Nigeria.

## Supplementary Information

**Additional file 1.**

**Additional file 2.**

**Additional file 3.**

## Data Availability

The data set for this study are available as supplementary material.

## Abbreviations

AIP: Atherogenic Index of Plasma
BMI: Body Mass Index
CHEWs: Community Health Extension Workers
CHOs: Community Health Officers
CVD: Cardiovascular Disease
CVDs: Cardiovascular Diseases
DBP: Diastolic Blood Pressure
FBG: Fasting Blood Glucose
HDL: High Density Lipoproteins
HDL-C: High Density Lipoproteins Cholesterol
IBM/SPSS: International Business Machines Corporation/ Statistical Package for the Social Sciences
IQR: Interquartile range
JNC: Joint National Committee
LDL: Low Density Lipoproteins
LDL-C: Low Density Lipoproteins Cholesterol
LICs: Low-Income Countries
LMICs: Low-and-Middle-Income Countries
NCDs: Non-Communicable Diseases
PE: Pulmonary Embolism
PHC: Primary Health Care/
SBP: Systolic Blood Pressure
SSA: Sub-Saharan Africa
TAG/Tg: Triglycerides/
TC: Total Cholesterol
WHO: World Health Organization
