# Supplementary material for: The ten-year risk of developing cardiovascular disease among public health workers in North-Central Nigeria using Framingham and atherogenic index of plasma risk scores
Source: BMC Public Health. 2022 Apr 27;22:847. doi: 10.1186/s12889-022-13044-9 (PMC9047388; doi:10.1186/s12889-022-13044-9)
Supplement: Supplementary file 2 — Additional file 2. [file 12889_2022_13044_MOESM2_ESM.docx]

# INFORMATION SHEET

**THE TEN-YEAR RISK OF DEVELOPING CARDIOVASCULAR DISEASE AMONG PUBLIC HEALTH WORKERS IN NORTH-CENTRAL NIGERIA USING FRAMINGHAM AND ATHEROGENIC INDEX OF PLASMA RISK SCORES**

**RESEARCHER**I am Dr Olubiyi O.A, a medical doctor. I am conducting research on **The Ten-Year risk of Developing Cardiovascular Disease among Public Health Workers in North-Central Nigeria using Framingham and Atherogenic Index of Plasma Risk Scores**

The purpose of this study is to determine the ten-year risk of developing cardiovascular disease among health workers in public health services in North-Central Nigeria.

The study procedure will entail collection of data on socio-demographics, cardiovascular disease risk knowledge and practices. There will also be measurement of weight, height, waist and blood pressure. Blood samples will also be taken for blood glucose and lipid profile determination.

**RISKS**
There are minimal risks to this study. The risk includes some discomfort while taking measurements and laboratory sample.

**BENEFITS**

You will benefit by improving your knowledge on CVD risks and prevention practices. You will also be afforded the opportunity to be screened for high blood pressure, obesity, dyslipidaemia, diabetes.

**CONFIDENTIALITY**

Your responses to this study will be anonymous. Please do not write any identifying information on your questionnaire. Every effort will be made by me to preserve your confidentiality by assigning code names/numbers for participants that will be used on all research notes and documents.

**CONTACT INFORMATION**

If you have questions at any time about this study, you may contact me.

**VOLUNTARY PARTICIPATION**

Your participation in this study is voluntary. It is up to you to decide whether or not to take part in this study. If you decide to take part in this study, you will be asked to sign a consent form. After you sign the consent form, you are still free to withdraw at any time and without giving a reason

**CONSENT**
I have read, and I understand the provided information and have had the opportunity to ask questions. I understand that my participation is voluntary and that I am free to withdraw at any time, without giving a reason and without cost. I understand that I will be given a copy of this consent form. I voluntarily agree to take part in this study.

Participant's Signature ______________________________ Date ________________

Investigator's Signature _____________________________ Date ________________

**NOTE:** The interviewer should screen the participants for the following before starting the interview;

1. Respondent should have consented to undergo this study
2. Should not have obvious cardiovascular disease
3. Should not have any debilitating illness

# QUESTIONNAIRE

**The Ten-Year risk of Developing Cardiovascular Disease among Public Health Workers in North-Central Nigeria using Framingham and Atherogenic Index of Plasma Risk Scores**

**Respondents ID: ………………………………………….**

**Facility ID: …………………………………………..**

**Name of interviewer: ……………………………………..**

**Date of interview: ...………………………………………..**

**SECTION A: SOCIO-DEMOGRAPHY**

**1. Age:** **|__|__|__|** years Unknown

If unknown: estimated Current Age: **|__|__|__|** years

**2. Sex**: Male Female

**3. What is your present marital status?**

Single Married Separated Divorced Widowed

**4. What is your** **Ethnic group?**

Nupe Hausa Yoruba Igbo Other, specify ___________________

**5. Please describe your main job during the last 12 months:**

Doctor Nurse Pharmacist CHEW/CHO Laboratory Scientist/Technician Other, specify_____________________

**6**. **Which department do you work in? please specify_______________**

**7. What is your level of education?**

Diploma Bachelors Masters PhD Postgraduate Diploma Medical fellowship Other, specify_________________________

**8. What is your religion?**

None Christianity Islam Traditional African Religion Other, specify_______________________

**9. What is the estimated total income per month?** (Round- to whole naira)_____________ **NAIRA**

**SECTION B: KNOWLEDGE OF CARDIOVASCULAR RISKS**

| **KNOWLEDGE** | Yes | No | I Don’t know |
| --- | --- | --- | --- |
| **CVD risks** |  |  |  |
| 10. Heart disease can be prevented |  |  |  |
| 11. Cigarette smoking can cause heart disease and/ or stroke |  |  |  |
| 12. There is greater risk for heart disease in the elderly |  |  |  |
| 13. Salty food causes high blood pressure |  |  |  |
| 14. If people quit smoking the risk of heart disease is reduced |  |  |  |
| 15. People with high blood pressure should avoid salt in their diets |  |  |  |
| 16. High blood pressure is a risk factor for heart disease |  |  |  |
| 17. Fatty foods increase blood cholesterol levels |  |  |  |
| 18. Eating more than 3 meals containing red meat per week is not good for your health |  |  |  |
| 19. Eating fruits and vegetables every day is beneficial |  |  |  |
| 20. Overweight people are more likely to have heart disease |  |  |  |
| 21. Regular exercise reduces the risk of heart disease |  |  |  |
| 22. Sadness can increase the risk of heart disease |  |  |  |
| 23. Stress increase the risk of heart disease |  |  |  |
| 24. Under stressful situations, blood pressure will increase |  |  |  |
| 25. Heavy alcohol use affects blood pressure |  |  |  |
| 26. High cholesterol is a risk factor for heart disease |  |  |  |
| 27. Diabetes is a risk factor for heart disease |  |  |  |
| 28. Heart disease in one’s family will increase one’s risk of heart disease |  |  |  |
| **CVD symptoms** |  |  |  |
| 29. Shortness of breath may be a sign of heart disease |  |  |  |
| 30. Feeling chest pain or discomfort can be a sign of heart disease |  |  |  |
| **CVD prevention, control and treatment** |  |  |  |
| 31. Controlling blood pressure reduces the risk of heart disease |  |  |  |
| 32. People with high blood pressure need to use blood pressure medicine for life |  |  |  |
| 33. People with high cholesterol despite diet and exercise need to take medication |  |  |  |
| 34. People with diabetes need to control their blood sugar |  |  |  |
| 35. People should only get their blood pressure checked if they have chest pain or headaches |  |  |  |
| 36. If you have high blood pressure taking a medicine for 1 month can cure you |  |  |  |

**37. What is your main source of health information?**

Television Radio Newspaper Internet Health care worker Friend/Relative/Opinion Leader Others, specify ___________________

**SECTION C: RISK FACTORS FOR CARDIOVASCULAR DISEASE**

**38.** **Are you taking drugs for any of the following conditions?**

Hypertension Yes No

Diabetes Yes No

High cholesterol Yes No

**39. Are you obese** Yes No

**40.** **Does anybody in your family suffer any of the following conditions?**

Hypertension Yes No

Diabetes Yes No

Stroke Yes No

Sudden death Yes No

Heart failure Yes No

**SECTION D: CARDIOVASCULAR DISEASE RISK PREVENTION PRACTICES**

**41. Do you smoke cigarette?**  If **never**, skip to **Q.44**

Never Ex-smoker < 1 year Ex-smoker ≥1 year Current smoker

**42.** **If ever, how many sticks per day?**

1 in 3 days 1-3 daily ≥4 daily

**43.** **Why do you smoke?** _____________________________________________

**44.** **Do you drink alcohol?** If **no** skip to **Q.46**

Yes No

**45a.**  **If yes, why do you drink? ______________________________________________**

**b. What type of alcohol do you take Beer Gin Whisky Vodka Palmwine Local gin**

**c. Quantify the volume of alcohol taken daily___________________________________**

**46. a**  **Do you engage in physical activity** *(e.g. walking, jogging, swimming, etc)*

Yes No

b**. if yes, what type of exercise?** Walking Jogging swimming Others (specify)

**47. If yes, how often do you engage in the physical activity per week?**

Occasionally 1-2 days 3-5 days every day

**48. What is the average time you use for the physical exercise per day?**

**<** 30 minutes **≥** 30 minutes

**49.** **How often did you add salt to food while cooking?**

Always Usually Sometimes Rarely Never

**50. How often did you add salt to any food at the table?**

Always Usually Sometimes Rarely Never

**51. Where is the food you eat mostly prepared?**

At Home Restaurant/Food vendor Place of work

**52. Does your job involve eating out of home a lot?**

Yes No

**53. How often did you eat food that was fried at home?**

Daily 4-6 times a week 1-3 times a week Less than once a week Never

**54. How often did you eat fried food away from home?**

Daily 4-6 times a week 1-3 times a week Less than once a week Never

**55. What did you do with the visible fat on your meat?**

Ate most of the fat Ate some of the fat Ate as little as possible Did not eat meat at all

**56. What did you do with the skin of your chicken or turkey?**

Ate most of the skin Ate some of the skin Ate as little as possible Did not eat chicken or turkey at all

**57. How would you describe your appetite?**

Very poor Poor Average Good Very good

**58. Are you frequently under stressed conditions or on-the-move?**

Yes No

**59. How often do you check your blood pressure**

Once a week Twice a month Once a month Once in six months Once a year Occasionally Never Others (Please specify) ______________________________

**60. When last did you check your blood pressure**

Last week Last month Last six months Last year More than a year (Please specify value)______________________________________________

**61. How often do you check your blood glucose level**

Once a week Twice a month Once a month Once in six months Once a year Occasionally Never Others (Please specify) ______________________________

**62. When last did you check your blood glucose level?**

Last week Last month Last six months Last year More than a year (Please specify value)______________________________________________

**63. How often do you check your blood lipid level**

Once a week Twice a month Once a month Once in six months Once a year Occasionally Never Others (Please specify) ______________________________

**64. When last did you check your blood lipid level?**

Last week Last month Last six months Last year More than a year (Please specify value)______________________________________________

**65. Can you recall your diet in the last 24 hours ______________________________**

Please answer every question. If you are uncertain about how to answer a question then do the best you can, but please do not leave a question blank.

**63. Your diet last 24 hours**

For each food there is an amount shown, either as a ‘medium serving’ or a common household unit, such as a slice or teaspoon. Please put a tick (🗸) in the box to indicate how often, **on average**, you have eaten the specified amount of each food **during the past 24 hours.**

| **Foods and amounts Average use** | | | | |
| --- | --- | --- | --- | --- |
| **Carbohydrate**  (medium serving) | Once a day | 2-3 per day | 4-5 per day | 6+ per day |
| Yam, Amala |  |  |  |  |
| Cassava (garri, fufu) |  |  |  |  |
| Plantain |  |  |  |  |
| Bread |  |  |  |  |
| Rice |  |  |  |  |
| Pasta e.g. spaghetti |  |  |  |  |
| Corn, cereal, Pap |  |  |  |  |
| Potato-sweet |  |  |  |  |
| Wheat, semo vita, flour |  |  |  |  |

| **Foods and amounts** |  |  |  |  |
| --- | --- | --- | --- | --- |
| **Protein**  (medium serving) | Once a day | 2-3 per day | 4-5 per day | 6+per day |
| Beans ( ekuru, moimoi, akara) |  |  |  |  |
| Fish (dried, fresh, iced) |  |  |  |  |
| Egg (whole, fried) |  |  |  |  |
| White meat (chicken, turkey, snail) |  |  |  |  |
| Cheese |  |  |  |  |
| Milk ( all types) |  |  |  |  |
| Red meat (goat, cow) |  |  |  |  |
| **Foods and amounts** |  |  |  |  |
| **Fruits and Vegetables**  (1 fruit or slice) | Once a day | 2-3 per day | 4-5 per day | 6+per day |
| Mango |  |  |  |  |
| Orange |  |  |  |  |
| Banana |  |  |  |  |
| Pawpaw |  |  |  |  |
| Apple |  |  |  |  |
| Water melon |  |  |  |  |
| Cucumber |  |  |  |  |
| Garden egg |  |  |  |  |
| Pineapples |  |  |  |  |
| Cashew |  |  |  |  |
| Carrots |  |  |  |  |
| Soup ingredients e.g. tomato, onion, okro, mushrooms (serving) |  |  |  |  |
| **Foods and amounts** |  |  |  |  |
| **Fat and Oil**  (tablespoon) | Once a day | 2-3 per day | 4-5 per day | 6+per day |
| Oil/fat, animal fats |  |  |  |  |
| Butter (teaspoon) |  |  |  |  |
| Coconut oil |  |  |  |  |
| Palm oil |  |  |  |  |
| Groundnut oil |  |  |  |  |

| **Foods and amounts** |  |  |  |  |
| --- | --- | --- | --- | --- |
| **Sweets and Snacks** (medium serving) | Once a day | 2-3 per day | 4-5 per day | 6+ per day |
| Sweet biscuit (one) |  |  |  |  |
| Cakes |  |  |  |  |
| Ice cream |  |  |  |  |
| Snacks e.g. pies, buns, doll nuts, puff-puff |  |  |  |  |
| Crisps or other packet snacks |  |  |  |  |
| Peanuts or other nuts |  |  |  |  |
| Sweets, toffee, mints |  |  |  |  |
| Sugar added to tea, coffee, cereals (teaspoon) |  |  |  |  |

| **Foods and amounts** |  |  |  |  |
| --- | --- | --- | --- | --- |
| **Drinks** | Once a day | 2-3 per day | 4-5 per day | 6+ per day |
| Tea (cup) |  |  |  |  |
| Coffee (cup) |  |  |  |  |
| Beverage e.g. bourn vita, cocoa vita, ovaltine, milo (cup) |  |  |  |  |
| Wine (glass) |  |  |  |  |
| Beer (bottle) |  |  |  |  |
| Spirits e.g. gin, brandy, whisky (shingle) |  |  |  |  |
| Low calorie or diet fizzy soft drinks (glass) |  |  |  |  |
| Fizzy soft drinks e.g.coca cola, pepsi (glass) |  |  |  |  |
| Pure fruit juice (100%) e.g. orange, apple juice (glass) |  |  |  |  |

**Section E: Reasons Influencing Adherence to Cardiovascular Risk Prevention**

**64. Do you have a BP monitor?** Yes No

**65. a. Do you have difficulties doing exercise?** Yes No

**b. If yes, what is the extent of difficulties that you have during exercise?** Minimum Low Moderate High

**c. Specify the type of difficulty _____________________________**

**d. If No, what are the barriers to exercise?** Feeling tired Busy schedule Pain Shortness of breath weather condition locations away from home lack of exercise partner Other please specify ________________________

**66.** **a. Do you eat healthy?** Yes No

b**. If No, what are the barriers to eating healthy?** Eating out (restaurant, ceremonies, work, family & friends homes) Inappropriate dietary habits (e.g. eating snacks in-between meals) Financial constraints (to procure idea healthy diets) Poor self control

**67. What are the reasons for not eating healthy always?** Granting self permission (e.g. just this once, a little won’t hurt) When in other peoples’ Situations at home Other please specify_______________________________________________________

**68. Are you stressed?** Yes No

**b. If yes, what is your coping method?** Repression being silent Nervous angry screaming shouting destroying objects Other please specify **_________________**

**67. Do you have social support?**  Yes  **N**o

#

# APPENDIX VI: PHYSICAL EXAMINATION AND LABORATORY REPORT FORMS

**Physical Examination Record**

1. Weight **|__|__|__| . |__|** kg
2. Height **|__|__|__| . |__|** cm
3. BMI **|__|__| . |__|** kg/m^2^
4. Blood pressure
5. **|__|__|__| / |__|__|__|** mmHg
6. **|__|__|__| / |__|__|__|** mmHg
7. Average blood pressure: **|__|__|__| / |__|__|__|** mmHg
8. Waist circumference **|__|__|__| . |__|** cm

**Laboratory Report Form**

| **Test (mmol/L)** | **Date** | **Result** |
| --- | --- | --- |
| Total- C | ­ |  |
| HDL-C |  |  |
| LDL-C |  |  |
| Triglycerides |  |  |
| Serum Glucose |  |  |

# APPENDIX VII: FRAMINGHAM RISK SCORE CHART

|  | 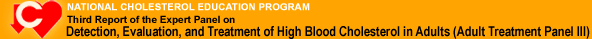 |  |
| --- | --- | --- |
|  | Top of Form     \| \| **Risk Assessment Tool for Estimating 10-year Risk of Developing Hard CHD (Myocardial Infarction and Coronary Death)** \| \| \| --- \| --- \| \| The risk assessment tool below uses recent data from the Framingham Heart Study to estimate 10-year risk for “hard” coronary heart disease outcomes (myocardial infarction and coronary death). Use the calculator below to estimate risk. \| \| \| Age: \| years \| \| Gender: \| Female Male \| \| [Total Cholesterol:](http://hp2010.nhlbihin.net/atpiii/calculator.asp?usertype=prof#cholesterol) \| mg/dL \| \| [HDL Cholesterol:](http://hp2010.nhlbihin.net/atpiii/calculator.asp?usertype=prof#hdl) \| mg/dL \| \| [Smoker:](http://hp2010.nhlbihin.net/atpiii/calculator.asp?usertype=prof#smoker) \| No Yes \| \| [Systolic Blood Pressure:](http://hp2010.nhlbihin.net/atpiii/calculator.asp?usertype=prof#sbp) \| mm/Hg \| \| Currently on any medication to treat high blood pressure. \| No Yes \| \|  \| \| \| \| --- \| --- \| --- \| --- \| --- \| --- \| --- \| --- \| --- \| --- \| --- \| --- \| --- \| --- \| --- \| --- \| --- \| --- \| --- \| --- \| --- \|   Bottom of Form   \|  \|  \| \| --- \| --- \| \|  \|  \| \|  \|  \| \|  \|  \| \|  \|  \| |  |
|  |  |  |
