# Supplementary material for: The ten-year risk of developing cardiovascular disease among public health workers in North-Central Nigeria using Framingham and atherogenic index of plasma risk scores
Source: BMC Public Health. 2022 Apr 27;22:847. doi: 10.1186/s12889-022-13044-9 (PMC9047388; doi:10.1186/s12889-022-13044-9)
Supplement: Supplementary file 3 — Additional file 3. [file 12889_2022_13044_MOESM3_ESM.docx]

**Table 1:** describes the socioeconomic characteristics of the health workers in the study area. It reflects the age, gender, cadre of the workers, their level of education, income and facilities they worked for.

| **Socioeconomic characteristics** | **Frequency (N=301)** | **%** |
| --- | --- | --- |
| **Age (years)** |  |  |
| 21 – 30 | 54 | 17.9 |
| 31 – 40 | 115 | 38.3 |
| 41 – 50 | 100 | 33.2 |
| 51 – 60 | 32 | 10.6 |
| Mean (± SD) | 39.30 (± 8.30) |  |
| Range | 22 – 58 |  |
|  |  |  |
| **Sex** |  |  |
| Male | 141 | 46.8 |
| Female | 160 | 53.2 |
|  |  |  |
| **Cadre** |  |  |
| Doctor | 41 | 13.6 |
| Nurse | 205 | 68.1 |
| Pharmacist | 9 | 3.0 |
| CHEW/CHO | 30 | 10.0 |
| Laboratory Scientist/tech | 16 | 5.3 |
|  |  |  |
| **Health Facility** |  |  |
| PHC | 27 | 9.0 |
| Secondary | 73 | 24.2 |
| Tertiary | 201 | 66.8 |
|  |  |  |
| **Level of education** |  |  |
| Diploma | 129 | 42.9 |
| Bachelors | 129 | 42.9 |
| Postgraduate | 43 | 14.2 |
|  |  |  |
| **Income (₦)** |  |  |
| ≤ 100,000 | 80 | 26.6 |
| 101,000 - 200,000 | 128 | 42.5 |
| 201,000 - 300,000 | 60 | 19.9 |
| > 300,000 | 33 | 11.0 |
| Median | 152,000.00 | |
| Interquartile range | 100,000.00 – 250,000.00 | |

The age of the respondents ranged between 21-58 years with a mean age of 39.3 years while the modal age group was 31-40 years. More than half, 160 (53.2%) of the respondents were females.

About two-thirds of the participants, 205(68.1%) were nurses and 201 (66.8%) work at the tertiary institution. Majority of the participants have either diploma or bachelors’ degree (42.9% respectively). The median income in Naira per month was **₦**152,000 with an interquartile range of **₦**100, 000-250,000.

**Table 2:** illustrates the Framingham and Atherogenic Index of Plasma risk score grading of the health workers. It describes the proportion of health workers with their level of risk using both Framingham risk score and atherogenic index of plasma score.

| **Risk scoring** | **Frequency (N=301)** | **%** |
| --- | --- | --- |
| **Framingham risk score** |  |  |
| Low risk | 296 | 98.3 |
| Moderate risk | 3 | 1.0 |
| High risk | 2 | 0.7 |
| **Atherogenic Index of Plasma** |  |  |
| Mild risk | 281 | 93.4 |
| Intermediate | 14 | 4.7 |
| High risk | 6 | 2.0 |

Following the grading of the Framingham risk scores, majority of the health workers, 296 (98.3%) have low 10-year risk of developing cardiovascular disease. Likewise, after grading the Atherogenic Index of Plasma scores, majority of the health workers, 281 (93.4%) have low risk of developing CVD from dyslipidaemia.

**Table 3:** shows the relationship between the fasting lipid profile and atherogenic index of plasma of the health workers and their job cadre. It reflects the risks associated with the job cadre of the health workers.

|  | | **Job cadre** | | | | | | | | | | | |  | |  |
| --- | --- | --- | --- | --- | --- | --- | --- | --- | --- | --- | --- | --- | --- | --- | --- | --- |
|  | | **Doctor** | | **Nurse** | | **Pharm** | | **CHEW** | **Lab** | | | **Total** | | **χ^2^** | | ***p* value** |
| **Variable** | | **n (%)** | | **n (%)** | | **n (%)** | | **n (%)** | **n (%)** | | | **N** | |  | |  |
| **T.C** | |  | |  | |  | |  |  | | |  | |  | |  |
| Optimal | | 15(36.6) | | 69(33.7) | | 2(22.3) | | 16(53.3) | 10(62.5) | | | 112(37.2) | | 11.235^Y^ | | 0.188 |
| Borderline | | 15(36.6) | | 80(39.0) | | 3(33.3) | | 4(13.4) | 2(12.5) | | | 104(34.6) | |  | |  |
| High risk | | 11(26.8) | | 56(27.3) | | 4(44.4) | | 10(33.3) | 4(25.0) | | | 85(28.2) | |  | |  |
| **HDL** | |  | |  | |  | |  |  | | |  | |  | |  |
| High risk | | 1(2.4) | | 16(7.8) | | 0(0.0) | | 2(6.7) | 3(18.8) | | | 22(7.3) | | 4.128^Y^ | | 0.845 |
| Beneficial | | 2(4.9) | | 21(10.2) | | 1(11.1) | | 2(6.7) | 0(0.0) | | | 26(8.6) | |  | |  |
| **LDL** | |  | |  | |  | |  |  | | |  | |  | |  |
| Optimal | | 30(73.2) | | 150(73.2) | | 8(88.8) | | 21(70.0) | 12(75.0) | | | 221(73.4) | | 3.199^Y^ | | 0.999 |
| Borderline | | 6(14.6) | | 26(12.7) | | 1(11.1) | | 6(20.0) | 2(12.5) | | | 41(13.6) | |  | |  |
| High risk | | 5(12.2) | | 29(14.2) | | 0(0.0) | | 3(10.0) | 2(12.5) | | | 40(13.0) | |  | |  |
| **Triglyceride** | |  | |  | |  | |  |  | | |  | |  | |  |
| Optimal | | 38(92.7) | | 181(88.3) | | 9(100.0) | | 26(86.7) | 16(100.0) | | | 270(89.7) | | 1.458^Y^ | | 0.993 |
| Borderline | | 1(2.4) | | 15(7.3) | | 0(0.0) | | 3(10.0) | 0(0.0) | | | 19(6.3) | |  | |  |
| High risk | | 2(4.9) | | 9(4.4) | | 0(0.0) | | 1(3.3) | 0(0.0) | | | 12(4.0) | |  | |  |
|  | |  | |  | |  | |  |  | | |  | |  | |  |
| **AIP** | |  | |  | |  | |  |  | | |  | |  | |  |
| Mild risk | | 41(100.0) | | 187(91.3) | | 91(100.0) | | 28(93.4) | 16(100.0) | | | 281(93.4) | | 3.160Y | | 0.923 |
| Intermediate | | 0(0.0) | | 13(6.3) | | 0(0.0) | | 1(3.3) | 0(0.0) | | | 14(4.7) | |  | |  |
| High risk | | 0(0.0) | | 5(2.4) | | 0(0.0) | | 1(3.3) | 0(0.0) | | | 6(2.0) | |  | |  |
|  |  | |  | |  | |  | |  |  |  | |  | |  |  |

**χ^2^: Chi square test; Y: Yates corrected Chi square; *: *p* value <0.05; Pharm: Pharmacists; Lab: Laboratory scientist/technician**

There was no statistically significant association between the fasting lipid profile as well as the atherogenic index of plasma of the health workers and their job cadre.

**Table 4:** shows the relationship between knowledge of cardiovascular disease risk and the clinical risk scoring of the health workers using Yates corrected Chi-square test of association.

|  | **Knowledge** | | |  |  |
| --- | --- | --- | --- | --- | --- |
| **Clinical risk scoring** | **Good** | **Poor** | **Total** | **χ^2^** | ***p* value** |
|  | **n (%)** | **n (%)** | **N** |  |  |
| **Framingham risk score grade** |  |  |  |  |  |
| Low risk | 287 (97.0) | 9 (3.0) | 296 | 5.289^Y^ | 0.071 |
| Moderate risk | 3 (100.0) | 0 (0.0) | 3 |  |  |
| High risk | 2 (100.0) | 0 (0.0) | 2 |  |  |
| **Atherogenic Index of Plasma** |  |  |  |  |  |
| Mild risk | 272 (96.8) | 9 (3.2) | 281 | 0.608^Y^ | 0.738 |
| Intermediate | 14 (100.0) | 0 (0.0) | 14 |  |  |
| High risk | 6 (100.0) | 0 (0.0) | 6 |  |  |

**χ^2^: Chi square test; Y: Yates corrected Chi square.**

There was no statistically significant association between good knowledge of cardiovascular disease and Framingham risk score and AIP dyslipidaemia risk score. (p>0.05)

**Table 5:** shows the relationship between practice of cardiovascular disease prevention and clinical risk scoring using Yates corrected Chi-square test of association.

|  | **Practice** | | |  |  |  |
| --- | --- | --- | --- | --- | --- | --- |
| **Clinical risk scores** | **Poor** | **Fair** | **Good** | **Total** | **χ^2^** | ***p*-value** |
|  | **n (%)** | **n (%)** | **n (%)** | **N (%)** |  |  |
| **Framingham** |  |  |  |  |  |  |
| Low risk | 37 (12.5) | 202 (68.2) | 57 (19.3) | 296 (98.3) | 0.474^Y^ | 0.976 |
| Moderate risk | 0 (0.0) | 2 (66.7) | 1 (66.7) | 3 (1.0) |  |  |
| High risk | 0 (0.0) | 1 (50.0) | 1 (50.0) | 2 (0.7) |  |  |
| **Atherogenic Index of Plasma** |  |  |  |  |  |  |
| Mild risk | 34 (12.1) | 191 (68.0) | 56 (19.9) | 281 (93.4) | 0.261^Y^ | 0.992 |
| Intermediate | 2 (14.3) | 10 (71.4) | 2 (14.3) | 14 (4.7) |  |  |
| High risk | 1 (16.1) | 4 (66.7) | 1 (16.7) | 6 (2.0) |  |  |

**χ^2^: Chi square test;** **Y: Yates corrected Chi square**

There was no significant relationship between good CVD prevention practices and clinical risk scoring. (p values >0.05).

**Table 6:** reflects on the relationship between sex (male or female) and clinical risk of the health workers using Fisher’s exact test of association.

|  | **Sex** | | |  |  |
| --- | --- | --- | --- | --- | --- |
|  | **Male** | **Female** | **Total** | **χ^2^** | ***p* value** |
| **Variable** | **n (%)** | **n (%)** | **N (%)** |  |  |
| **Framingham risk score** |  |  |  |  |  |
| Low risk | 137 (97.2) | 159 (99.4) | 296 (98.3) | 3.293^F^ | 0.176 |
| Moderate risk | 3 (2.1) | 0 (0.0) | 3 (1.0) |  |  |
| High risk | 1 (0.7) | 1 (0.6) | 2 (0.7) |  |  |
| **AIP** |  |  |  |  |  |
| Mild risk | 130 (92.2) | 151 (94.4) | 281 (93.4) | 3.171^F^ | 0.210 |
| Intermediate risk | 6 (4.3) | 8 (5.0) | 14 (4.6) |  |  |
| High risk | 5 (3.5) | 1 (0.6) | 6 (2.0) |  |  |

**χ^2^: Chi square test; F: Fisher’s exact test; t: Independent Samples T test**

There is no statistically significant association between sex Framingham risk score and atherogenic index of plasma (AIP) score.

**Table 7:** describes the correlation between Atherogenic Index of Plasma scores and cardiovascular disease risk factors of respondents using Spearman’s correlation.

|  | **AIP** | |
| --- | --- | --- |
| **Risk factors** | **r** | ***p* value** |
| BMI | 0.118 | **0.041*** |
| Blood pressure | -0.001 | 0.991 |
| SBP | 0.043 | 0.459 |
| DBP | -0.014 | 0.815 |
| Waist circumference | 0.174 | **0.002*** |
| Total cholesterol | -0.028 | 0.627 |
| HDL | -0.558 | **<0.001*** |
| LDL | -0.215 | **<0.001*** |
| Triglyceride | 0.912 | **<0.001*** |
| Fasting blood glucose | 0.182 | **0.002*** |
| Framingham score | 0.011 | 0.851 |

**r: Spearman’s correlation coefficient rho; *: *p* value <0.05**

Although only 20 (6.7%) of the health workers had intermediate-high risk AIP dyslipidaemia, there was a positively higher correlation between AIP score and triglyceride (0.912) and this was significant at P value <0.001, while there was a negatively high correlation between AIP score and HDL cholesterol (-0.558) at p value of <0.001. AIP risk was also significantly positively correlated to BMI (0.118, p value 0.041), waist circumference (0.174, p value 0.002) and fasting blood glucose (0.182, p value 0.002); and negatively correlated to LDL cholesterol (-0.215, p value <0.001).
